# Increased afterload induces pathological cardiac hypertrophy: a new in vitro model

**DOI:** 10.1007/s00395-012-0307-z

**Published:** 2012-10-26

**Authors:** Marc N. Hirt, Nils A. Sörensen, Lena M. Bartholdt, Jasper Boeddinghaus, Sebastian Schaaf, Alexandra Eder, Ingra Vollert, Andrea Stöhr, Thomas Schulze, Anika Witten, Monika Stoll, Arne Hansen, Thomas Eschenhagen

**Affiliations:** 1 Department of Experimental Pharmacology and Toxicology, University Medical Center Hamburg-Eppendorf and DZHK (German Centre for Cardiovascular Research), partner site Hamburg/Kiel/Lübeck, Martinistraße 52, 20246 Hamburg, Germany; 2 Leibniz Institute for Arteriosclerosis Research, Münster, Germany

**Keywords:** Afterload enhancement, Cardiac hypertrophy, Cardiac metabolism, Cardiac tissue engineering, Endothelin receptor antagonist, Fibrosis

## Abstract

Increased afterload results in ‘pathological’ cardiac hypertrophy, the most important risk factor for the development of heart failure. Current in vitro models fall short in deciphering the mechanisms of hypertrophy induced by afterload enhancement. The aim of this study was to develop an experimental model that allows investigating the impact of afterload enhancement (AE) on work-performing heart muscles in vitro. Fibrin-based engineered heart tissue (EHT) was cast between two hollow elastic silicone posts in a 24-well cell culture format. After 2 weeks, the posts were reinforced with metal braces, which markedly increased afterload of the spontaneously beating EHTs. Serum-free, triiodothyronine-, and hydrocortisone-supplemented medium conditions were established to prevent undefined serum effects. Control EHTs were handled identically without reinforcement. Endothelin-1 (ET-1)- or phenylephrine (PE)-stimulated EHTs served as positive control for hypertrophy. Cardiomyocytes in EHTs enlarged by 28.4 % under AE and to a similar extent by ET-1- or PE-stimulation (40.6 or 23.6 %), as determined by dystrophin staining. Cardiomyocyte hypertrophy was accompanied by activation of the fetal gene program, increased glucose consumption, and increased mRNA levels and extracellular deposition of collagen-1. Importantly, afterload-enhanced EHTs exhibited reduced contractile force and impaired diastolic relaxation directly after release of the metal braces. These deleterious effects of afterload enhancement were preventable by endothelin-A, but not endothelin-B receptor blockade. Sustained afterload enhancement of EHTs alone is sufficient to induce pathological cardiac remodeling with reduced contractile function and increased glucose consumption. The model will be useful to investigate novel therapeutic approaches in a simple and fast manner.

**Electronic supplementary material:**

The online version of this article (doi:10.1007/s00395-012-0307-z) contains supplementary material, which is available to authorized users.

## Introduction

Aging of the population and better survival of patients affected by coronary heart disease have led to an increasing number of patients with chronic heart failure, the common end stage of virtually all cardiac diseases. Its prevalence in the general population is 1–2 %, but reaches more than 10 % in octogenarians [4]. Left ventricular hypertrophy is the most important antecedent risk factor for the development of heart failure [21]. Cardiac hypertrophy is clearly double-edged. Under exercise or pregnancy it is beneficial and referred to as physiologic, under sustained hypertension or aortic stenosis cardiac hypertrophy is pathologic. Both types of hypertrophy are characterized by an increase in cardiomyocyte size, but only pathological hypertrophy is associated with increased apoptosis and fibrosis as well as functional changes such as altered cellular Ca^2+^ homeostasis, ion channel remodeling, reduced contractile force and relaxation velocity, predisposing factors for malignant arrhythmia and heart failure [41]. Despite considerable efforts, the mechanisms underlying the differences between physiological and pathological hypertrophy remain incompletely understood.

The classical model to study cardiac hypertrophy has been transverse aortic constriction (TAC), in which the afterload of hearts is increased by banding of either the thoracic or the abdominal aorta [29]. TAC reliably results in cardiac hypertrophy, but functional outcomes such as heart failure can be variable. Further disadvantages of this method are its costs in terms of labor and money, the impossibility of investigating human myocyte biology, and limitations inherent to in vivo models. Specifically, it is difficult to differentiate direct load-induced alterations from systemic and humoral mechanisms. In vitro models of cardiac hypertrophy have been developed to overcome some of these limitations, e.g. neonatal rat cardiomyocytes stimulated by α-adrenergic agonists or endothelin-1 [11], or, when cultured on silicone membranes, by phasic or tonic stretch [19]. Cardiomyocytes in cell culture are isotropically oriented, which may limit their informative value, as cardiomyocytes in heart tissue are highly organized. Moreover, passive stretch mimics an increase in preload rather than afterload, the more frequent cause of cardiac hypertrophy. Finally, standard 2D monolayer cell cultures do not allow measurement of contractile function, an important parameter differentiating “physiological” from “pathological” hypertrophy.

The aim of the present study was to generate a novel in vitro model that exhibits a tissue-like arrangement of cardiomyocytes and faithfully monitors the effects of increased afterload on cardiac tissue. One of the key factors was to switch from conventional 2-dimensional cardiomyocyte culture to a 3-dimensional format, called fibrin-based engineered heart tissue (EHT) [12]. The effect of afterload enhancement was compared to pharmacological stimulation of EHTs.

## Methods

A detailed description of materials and methods can be found at Supplemental Material online.

### Generation of EHTs

EHTs were generated as previously described with minor modifications [12]. Namely, we increased cell concentration from 4.1 to 5.0 × 10^6^ cells/mL (+22 %), we omitted Matrigel in EHTs, and reduced the initial volume of a single EHT from 145 to 100 μL (−31 %). Briefly, ventricular heart cells (the atria were carefully excised) from neonatal Wistar and Lewis rats (postnatal day 0 to 3) were isolated by a fractionated DNase/Trypsin digestion protocol. This procedure was reviewed and approved by the Ethics Commission of the Medical Association of Hamburg. Rat ventricular heart cells, fibrinogen, thrombin and DMEM (2×, to match the volumes of fibrinogen and thrombin and thus ensuring isotonic conditions) were mixed and pipetted into molds, which were obtained by casting 2 % agarose (in PBS) around Teflon^®^ (polytetrafluoroethylene) spacers in a 24-well culture dish (Online Table I). After polymerization of fibrin (1–2 h), EHTs were transferable to new cell culture dishes filled with medium (Online Fig. I).

### Manufacturing silicone racks with adjustable post resistance

EHTs were generated in casting molds, each of them containing a pair of small silicone tubes. They were part of custom-made silicone racks (Sylgard^®^ 184, Dow Corning), each carrying four pairs of silicone tubes (Fisher Scientific 3100504; Online Fig. I). The lower apertures of the tubes were closed by silicone discs, which ameliorated adherence of EHTs and prevented intrusion of medium. The silicone tubes were elastic and obeyed Hooke’s law for springs. They formed a defined resistance for beating EHTs, which could be expressed as force or spring constant *k*, its value under baseline conditions being 0.95 N/m (Online Fig. II). Inserting a metal brace into the two silicone tubes increased the resistance opposed by the silicone posts by a factor of 12 (*k* = 11.5 N/m). Metal braces were handmade from stainless steel Nubryte^®^Wire 016 (GAC, size 016, length: 14′′). The conditions after insertion of metal braces are referred to as afterload enhanced (AE) in this study. The preload of EHTs was 0.8 mN (tension generated by the silicone posts in diastole; i.e. resting state).

### Cell culture conditions

EHTs were maintained in 37 °C, 7 % CO_2_, and 40 % O_2_ humidified cell culture incubators throughout experiments. EHT medium for the first 8 days of culture consisted of DMEM (Biochrom F0415), 10 % horse serum inactivated (Gibco 26050), 2 % chick embryo extract, 1 % penicillin/streptomycin (Gibco 15140), insulin (10 μg/mL, Sigma-Aldrich 857653), and aprotinin (33 μg/mL, Sigma-Aldrich A1153). Between day 8 and 13 medium was used with the same composition except for reduced horse serum content (4 %). Up to day 13 medium was changed three times per week, afterwards daily. From day 13 on EHTs were kept in serum-free medium, i.e. the above medium without horse serum plus triiodothyronine (*T*_3_, 0.5 ng/mL, European Commission—Joint Research Centre IRMM-469). Supplementation of low concentrations of hydrocortisone (50 ng/mL, Sigma-Aldrich H0888) from day 13 on increased spontaneous beating rate and thereby reduced experimental drop-out rates (due to lack of beating) to less than 5 %. EHTs started to beat coherently one week after casting. Thereafter EHTs were filmed every other day. The movies were analyzed by a customized software (CTMV) which automatically calculated force, frequency, fractional shortening, contraction time *T*_1_ and relaxation time *T*_2_ of each single EHT, as recently published (Fig. 7a, b; Online Movie I) [12].

On day 14 hypertrophic interventions started. For most experiments we studied four different groups in parallel (*n* = 4–6 per group, one 24-well culture dish). Afterload of EHTs was increased by inserting metal braces (AE)—a mere mechanical intervention—in one group. In two other groups hypertrophic growth was triggered by either phenylephrine (PE, (R)–(–)–Phenylephrine hydrochloride, Sigma-Aldrich P6126) at 20 μmol/L or endothelin-1 (ET-1, Sigma-Aldrich E7764) at 5 nmol/L. Most interventions were carried out for 7 days (except cell size measurements and whole genome gene expression analysis, which both were analyzed after 5 days). EHTs without metal braces and without pharmacological agents served as controls (Online Fig. III). Endothelin receptors were blocked for selected experiments in the AE group. Endothelin receptor antagonists (ET-RA) in a concentration well above IC_50_ were added to the medium 2 h before metal braces were inserted and medium for all subsequent medium changes also contained ET-RAs (Online Table II).

## Results

### Establishment of serum-free cell culture conditions for EHTs

**Fig. 1:**
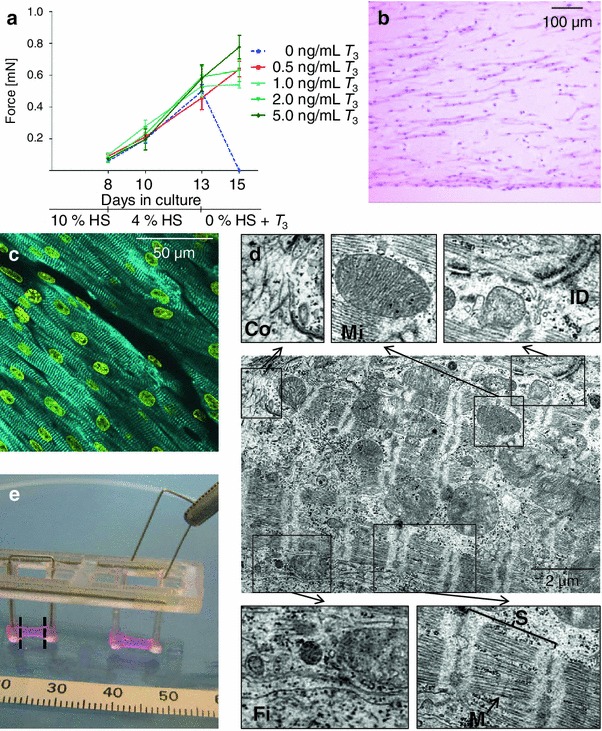
Formulation of serum-free EHT medium by *T*_3_ supplementation, experimental model of afterload enhancement. **a** 20 EHTs were cultured in identical medium for 13 days. Reduction of horse serum content on day 8 did not affect their development. From day 13 on EHTs were kept in medium free of horse serum, but in addition with varying *T*_3_ concentrations. Without *T*_3_ EHTs completely stopped to beat after 2 days (*dashed line*). Addition of *T*_3_ in a range of 0.5 to 5.0 ng/mL prevented the decline in force. **b–d** Microscopic images of an EHT cultured 8 days in horse-serum free and *T*_3_ 0.5 ng/mL supplemented medium. **b** H&E-stained paraffin section. **c** Merged confocal analysis of an area close to the surface of an EHT stained with an antibody against α-actinin (*cyan*) and with DRAQ5 (*yellow*). **d** Electron microscopy. Despite absence of serum for more than a week, there was a well-developed cardiac tissue structure with longitudinal alignment of cells and distinct ultrastructure of sarcomers with Z, I, A, H, and infrequently M-bands (*arrow*). *Fi* fibrin, *Mi* mitochondrion, *Co* collagen, *ID* part of an intercalated disc with desmosomes, *S* sarcomere, *M* M-Band. **e** Photograph of insertion of metal braces. EHTs and silicone racks were taken out of medium and cell culture dish only for better depiction, scale in millimeters. For histological or biomolecular analyses only the middle sections (between the *broken lines*) of EHTs were analyzed

The cell culture medium for EHTs contained 10 % horse serum in the first days after generation. After onset of definite coherent beating with post deflection, which usually occurred around day 8, the content of horse serum in the medium was reduced to 4 % for the following 5 days. This reduction did not affect the development of EHTs as demonstrated by a continuing increase in force, measured by repeated video-optical recordings (Fig. 1a). However, complete elimination of horse serum on day 13 lead to a rapid decline in beating activity and, not later than 48 h, to complete contractile inactivity. This effect could be prevented by addition of the thyroid hormone triiodothyronine (*T*_3_) to the medium. A range of *T*_3_ concentrations from 0.5 to 5.0 ng/mL was tested. No significant differences between these concentrations were detected, neither in force (Fig. 1a) nor in frequency (not shown). Even after 8 days without serum EHTs continued to beat (Online Movie I) and exhibited a loose, but interconnected and well-oriented cardiac tissue-like structure with longitudinal alignment of cross-striated cardiomyocytes in the middle regions of an EHT (Fig. 1b) and a tightly packed array of cardiomyocytes in the near-surface regions (Fig. 1c). Electron microscopy revealed regular sarcomeric ultrastructure, mitochondria of normal shape and size (mostly crista type, length around 2 μm), fibrin (the artificial extracellular matrix), and collagen (first primitive fibrils, Fig. 1d). As *T*_3_ at 0.5 ng/mL was not inferior to higher concentrations of *T*_3_ in terms of force development, frequency, and histology and as it was close to physiological values (Online Table III), it was used for all further experiments from day 13 on (Online Fig. III).

### Activation of the hypertrophic gene program

**Fig. 2:**
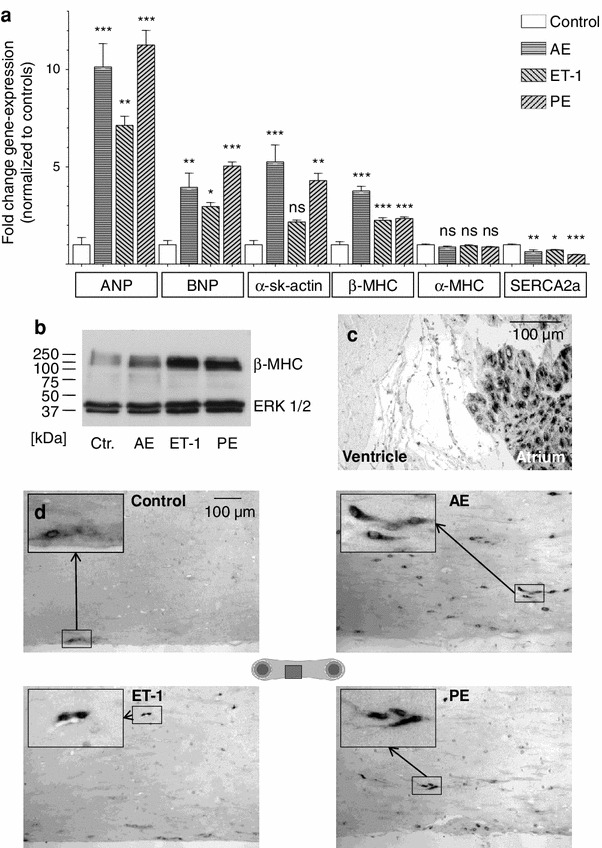
Activation of the hypertrophic gene program. **a** Transcript concentrations of members of the hypertrophic program as measured by RT-qPCR. All concentrations were normalized to controls. (*n* = 3–6 biological × 3 technical replicates). **b** Western blot analysis of EHTs (100,000 cells per lane) for β-MHC. The total amount of ERK 1/2 served as loading control. **c** Validation of ANP-immunohistochemistry on healthy adult rat heart. The characteristic perinuclear staining pattern for ANP could be detected in the atrium, whereas little ANP was detectable in the ventricle. **d** ANP-immunohistochemistry of EHTs. In control EHTs only very few cells at the surface stained positive for ANP. All EHTs subjected to hypertrophic interventions showed stronger ANP signals which were also perinuclearly located. The images cover more than one half of the EHT diameter (see pictograph) and were taken from the same area of the respective EHT

Insertion of metal braces into the hollow posts (Fig. 1e) increased the afterload of EHTs 12-fold and thus decreased post deflection, but it did not affect spontaneous beating rate (Online Movie II). In the control, the afterload enhanced (AE) and the ET-1-group we observed a pattern of contractions with 4 Hz and durations from 10–15 s alternating with resting periods of 10–20 s length (Online Movies I, II). PE-treatment shortened resting periods (Fig. 5c). To determine whether the three interventions (AE, ET-1, PE) induced the so-called hypertrophic gene program in EHTs under the experimental conditions chosen, transcript concentrations of atrial natriuretic peptide (ANP), brain natriuretic peptide (BNP), α-skeletal muscle isoform of actin (α-sk-actin), β-myosin heavy chain (β-MHC), α-myosin heavy chain (α-MHC), and SR Ca^2+^-ATPase 2 isoform b (SERCA2a) were measured in total RNA extracted from EHT homogenates by RT-qPCR (Fig. 2a). The strongest induction was observed for ANP. Its expression increased more than tenfold with AE (10.1×) or PE (11.3×), and slightly less with ET-1 (7.0×). In addition, BNP, α-sk-actin, and β-MHC were all induced by mechanical and by pharmacological stimulation. Transcript concentrations of SERCA2a, which is typically downregulated in cardiac hypertrophy, were also reduced by AE (0.64×), ET-1 (0.73×), or PE (0.49×). Expression of the major myosin isoform in adult rodents, α-MHC, was unaffected by the various stimulations. The four major isoforms of thyroid hormone receptors were downregulated in all three groups (not shown).

To corroborate our mRNA findings on the protein level, Western Blot analysis of β-MHC and immunohistochemistry of ANP were performed. β-MHC levels were low in control EHTs and robustly increased by AE and pharmacological stimulation (Fig. 2b). Immunohistochemistry staining conditions for ANP were optimized on adult rat heart tissue (Fig. 2c). The majority of atrial cardiomyocytes were ANP positive with a distinct perinuclear staining, whereas hardly any ANP signal was detectable in rat ventricles. Under the same staining conditions control EHTs showed weak and diffuse ANP signals only in scattered cells at the surface of the EHT. In AE-EHTs many cardiomyocytes throughout the tissue construct were ANP positive, also with the characteristic perinuclear staining. Distinct ANP positive cardiomyocytes were also observed in ET-1- and PE-treated EHTs, but preferentially at the free edges (Fig. 2d).

### Cardiomyocytes in EHTs enlarge with increased afterload or pharmacological stimulation

**Fig. 3:**
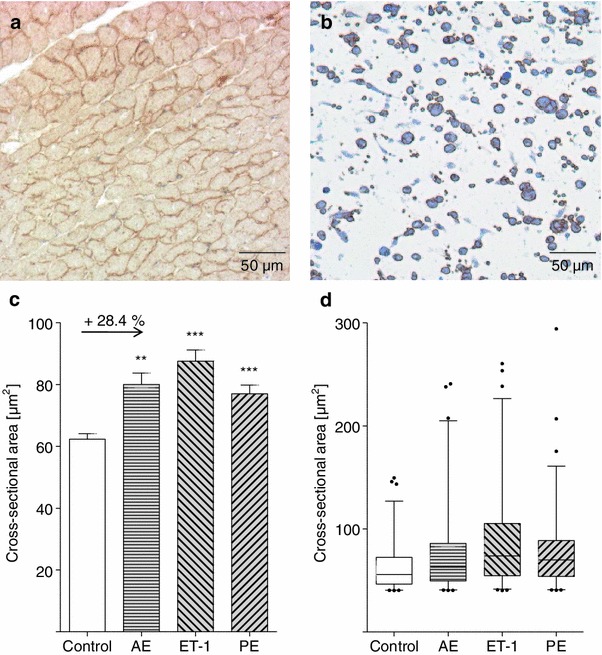
Specific cardiomyocyte cell membrane staining and in situ assessment of average cardiomyocyte size in EHTs. **a** Validation of dystrophin-immunohistochemistry on healthy adult rat heart. **b** Dystrophin-immunohistochemistry of an EHT cross section. Only cell membranes of cardiomyocytes were stained brown and thus distinguishable from other cell types. **c** Assessment of cross-sectional areas of 150 cardiomyocytes per group, three EHTs were analyzed per group. In control EHTs cardiomyocytes had a mean cross-sectional area of 62.3 μm^2^, after hypertrophic interventions mean sizes were 80.0 μm^2^ (afterload enhanced), 87.6 μm^2^ (ET-1-stimulated), and 77.0 μm^2^ (PE-stimulated). **d** Box-and-whisker diagram of cross-sectional area measurements. Whiskers represent percentile 2.5 and 97.5, dots display extreme values

Importantly, EHTs are generated from an unpurified population of neonatal rat ventricular heart cells and, analogous to real hearts, do not only contain cardiomyocytes, but also fibroblasts, endothelial cells and other native heart cells. To differentiate cardiomyocytes from the rest of cells and allow precise size determination, a specific dystrophin immunostaining was established on adult rat heart (Fig. 3a). Under optimized conditions, cardiomyocyte cell membranes were stained brown and other cell types, e.g. in blood vessels, remained unstained. In cross sections of EHTs cardiomyocytes were almost exclusively round in shape, indicating the high degree of longitudinal alignment in EHTs (Fig. 3b, see also Fig. 1b, c). Non-cardiomyocytes, visualized by a light counterstaining (blue), were devoid of the circular brownish staining, mostly smaller and more variable in shape. The distinct labeling of cardiomyocyte cell membranes permitted automated cross-sectional area measurements (Fig. 3c, d). This area was 62.3 μm^2^ on average in control EHTs, 80.0 μm^2^ (+28.4 %) in the AE-, 87.6 μm^2^ (+40.6 %) in the ET-1, and 77.0 μm^2^ (+23.6 %) in the PE-group. From the control to the hypertrophic groups all percentiles shifted to higher values indicating a hypertrophic effect on most cardiomyocytes in the EHTs (Fig. 3d).

### Unbiased analysis of alterations in gene expression and its comparison to TAC in mice

**Fig. 4:**
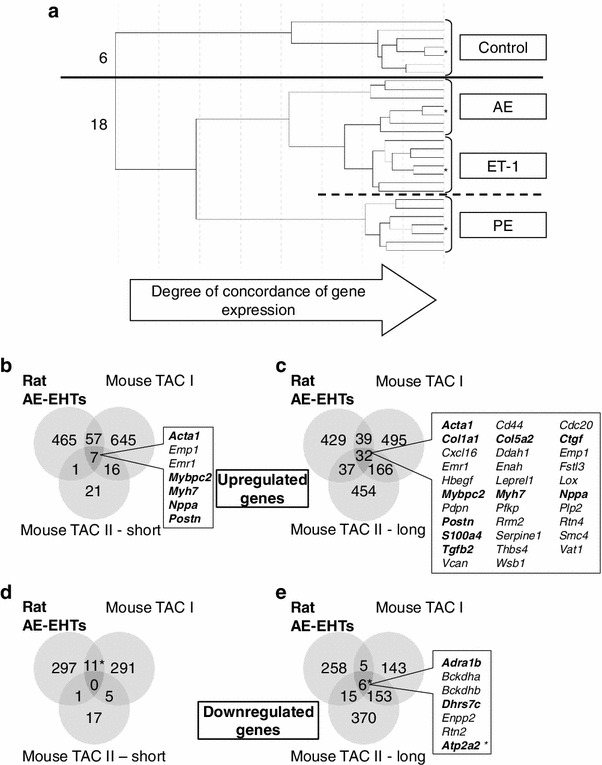
Gene expression analysis. **a** Software-aided clustering of 24 EHTs according to their concordance of gene expression. Input data was a whole transcriptome analysis (21,910 probes) for all EHTs. EHTs in the control group (*n* = 6 biological replicates, above the *thick line*) and in the groups with hypertrophic interventions (*n* = 18 biological replicates, below the *thick line*) were all classified correctly. *Asterisks* denote pairs of technical replicates. **b–c** Comparison of genes upregulated by afterload enhancement in EHTs (Rat AE-EHTs) with genes upregulated by TAC in mice. Mouse TAC I and II data sets were both taken from the literature. The latter data set was provided for two time points: short-term TAC (1 week, displayed in **b**, **d**) and long-term TAC (8 weeks, displayed in **c**, **e**). **d–e** Corresponding diagrams for downregulated genes in AE-EHTs and TAC. Note that Serca2a (*Atp2a2*) downregulation was falsely not detected by our rat microarray but by RT-qPCR. Its corrected positions are indicated with *asterisks*

Illumina^®^ microarray expression analysis covering almost 22,000 transcripts were used to obtain nearly whole genome gene expression information of EHTs. Each of the four conditions (control, AE, ET-1, PE) was analyzed in six biological replicates, creating 24 individual transcriptome data sets. The data sets were clustered by computer software according to their overall gene expression similarity (Fig. 4a). One sample of each group was assayed two times to determine technical reproducibility (asterisks). The later (i.e. the more on the right side) a path divided the more similar two EHTs were regarding their overall gene expression. The six controls formed one and the 18 other EHTs another distinct group, indicating precise division into control and hypertrophied EHTs at an early stage of the cluster analysis. The technical replicate in each group demonstrated the high degree of reproducibility of the expression chip and cluster analysis. Interestingly, clustering grouped AE and ET-1 together and apart from PE (broken line).

Compared to control EHTs between 572 and 1,405 genes were differentially expressed in the three hypertrophy groups. The fold change threshold for upregulation was set to 1.5, for downregulation to 0.66, accordingly. In detail, 648 genes were upregulated and 388 downregulated by AE. ET-1-stimulation upregulated 385 genes and downregulated 187 genes, PE induced 776 and repressed 629 genes. All typically upregulated genes in the hypertrophic gene program (see above) were represented in the list of the most strongly upregulated genes (Online Table IV). Notably, in the AE group 3 out of 4 typically upregulated hypertrophy-associated genes (*Myh7*, *Acta1*, *Nppa*) were found among the 5 most strongly upregulated genes (of overall 22,000 investigated genes). The fourth hypertrophic gene *Nppb* was ranked on position 60.

After conversion of up- or downregulated rat gene symbols in the AE group to mouse gene symbols they were compared to two mouse TAC data sets (Fig. 4 b–e): TAC I from Toischer et al. [37] was based on an Affymetrix^®^ expression array analysis, TAC II from Lee et al. [20] was obtained by RNA-Seq and provided data for short-term TAC (hypertrophic state) and long-term TAC (failing state). Seven genes (amongst others *Myh7*, *Acta1*, *Nppa)* were upregulated irrespective of species and analysis system in all three afterload enhancement procedures, i.e. AE-EHT, TAC I and short-term TAC II (Fig. 4b). The same analysis for long-term TAC II revealed more shared upregulated genes, e.g. extracellular matrix proteins and profibrotic or fibroblast markers (Fig. 4c). Genes collectively downregulated in AE, TAC I and long-term TAC II included *Adra1b* (alpha 1B adrenoceptor) and *Atp2a2* (Serca2a) (Fig. 4e).

An enrichment analysis revealed three molecular KEGG pathways in which genes upregulated in all 3 EHT hypertrophy groups (AE, ET-1, PE) were overrepresented: rno 04512 extracellular matrix, rno 03010 ribosome, and rno 00010 glycolysis. As transcriptional activation of ribosomal proteins seemed evident in a model of cardiac hypertrophy we subsequently focused on investigating glycolysis and extracellular matrix of EHTs under mechanical or pharmacological stimulation.

### Increased glycolysis in AE-EHTs

**Fig. 5:**
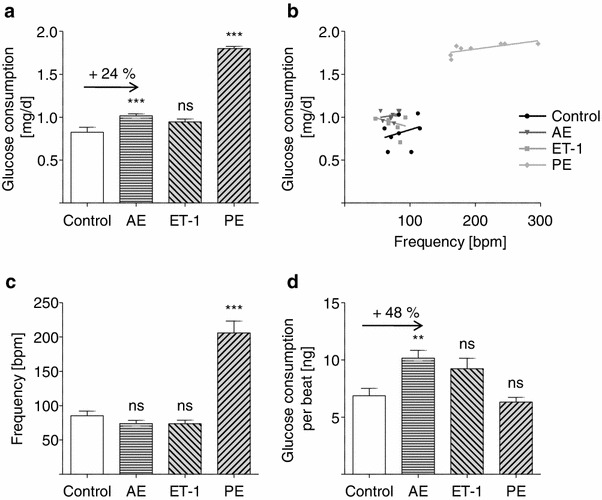
Glucose consumption. **a** Global glucose consumption of EHTs under control or hypertrophic conditions. **b** Glucose consumption correlated to some extent with beating rate of EHTs. The regression line of AE is shifted in *y*-direction compared to the regression line of control. Each point represents one EHT. **c** Spontaneous beating rate of control and hypertrophied EHTs. **d** Glucose consumption per beat as determined by dividing global glucose consumption by the calculated total number of beats over the observation period. (all *n* = 7–9 per group)

The identification of the KEGG pathway glycolysis in the enrichment analysis provided first evidence for an increase in glycolytic activity of AE-EHTs. Hence, the consumption of glucose—as the substrate of glycolysis —was directly measured (Fig. 5a). Under basal conditions one EHT consumed a mean of 0.83 mg glucose per day. Glucose consumption of AE-EHTs increased by 24 % to 1.02 mg/day (ET-1-EHTs: 0.94 mg/day, PE: 1.80 mg/day). Not surprisingly, glucose consumption correlated to some extent with the spontaneous beating rate, but on average AE-EHTs consumed more glucose at the same frequency than control EHTs (Fig. 5b). When averaged over time, frequencies of control, AE-EHTs and ET-1-EHTs did not differ, while PE-EHTs showed almost no resting periods and frequencies around 200 bpm (Fig. 5c). Glucose consumption amounted to 6.9 ng per beat in control EHTs and 10.2 ng in AE-EHTs (+48 %; Fig. 5d).

### Fibrosis in hypertrophied EHTs

Fibrosis is a hallmark of pathological cardiac hypertrophy and the fibrosis signals in the enrichment analysis and the comparison to mouse TACs prompted us to further investigate remodeling of EHTs after hypertrophic stimulation. Transcript concentrations of important structural and regulatory proteins of cardiac connective tissue were extracted from the whole-transcriptome analysis and verified by reverse transcription followed by quantitative PCR (Online Table V). Collagen-1 and collagen-3 are the major ECM components of the healthy heart and collagen-1 in particular is augmented in fibrotic hearts. In EHTs, transcript concentration of the collagen-1 pro-alpha chain *Col1a1* was increased by AE (1.55×), ET-1 (1.81×) and PE (1.33×). Fibronectin 1, a collagen linker protein, was also upregulated by AE (1.48×), ET-1 (2.16×) and PE (2.08×). In contrast, elastin and fibrillins, both elements of elastic fibers, were downregulated in all 3 hypertrophy groups: elastin (AE 0.40×; ET-1 0.51×; PE 0.12×) and fibrillin-1 (AE 0.55×; ET-1 0.64×; PE 0.51×). No changes were observed in the transcript concentrations of TGFβ1, in contrast to its downstream target connective tissue growth factor (CTGF), which was induced in all hypertrophy groups (AE 1.92×; ET-1 1.86×; PE 1.70×).

**Fig. 6:**
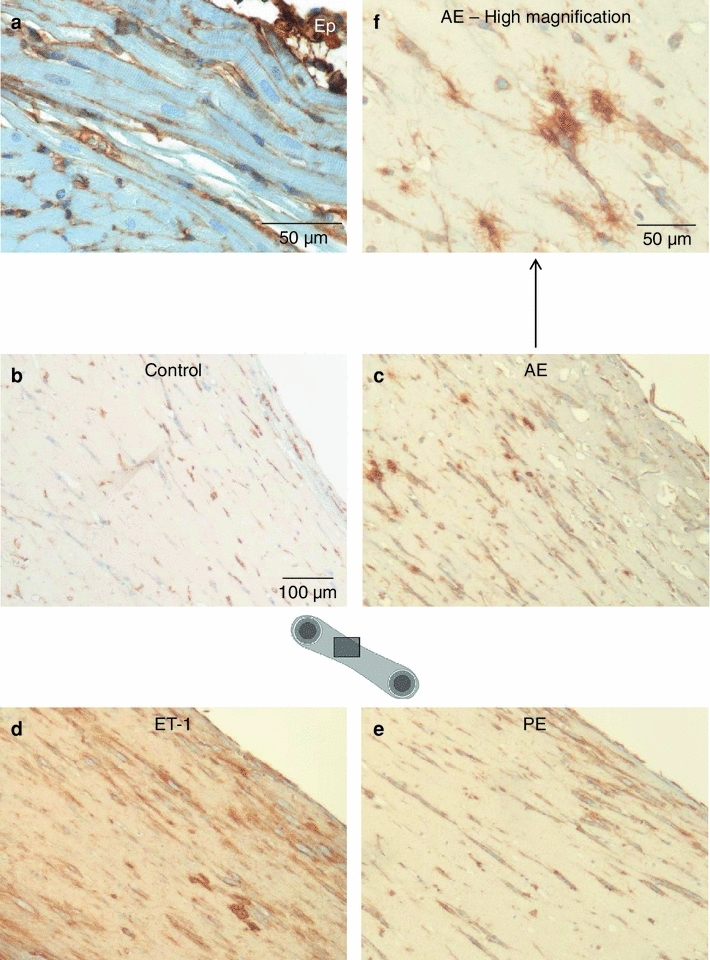
Collagen-1-immunohistochemistry as an indicator of fibrosis. **a** Validation of collagen-1-immunohistochemistry on paraffin sections of healthy adult rat heart. Collagen-1 was interspersed between cardiomyocytes and dominant in epicardium. **b** Low basal collagen-1 content in control EHTs in contrast to **c–e** interspersion with collagen-1 after hypertrophic interventions (all in *longitudinal* orientation). **f** Higher magnification of an afterload enhanced EHT. Fibrillar Collagen-1 protruded from cardiac fibroblasts and adsorbed onto cardiomyocytes

In order to investigate whether the increase in *Col1a1* transcripts corresponded to an increase in mature collagen-1 protein content, immunohistochemical staining was optimized with an antibody specific for a three-dimensional epitope of collagen-1 using adult rat heart as a positive control (Fig. 6a). Under the same staining conditions collagen-1 content appeared low in control EHTs (Fig. 6b), but showed a marked increase in all 3 intervention groups. Here, fibrillar collagen was found throughout the constructs (Fig. 6c–e), localized radially around cardiac fibroblasts and adsorbed onto cardiomyocytes (exemplarily in Fig. 6f). The remodeling process did not alter EHT-width in any group but it shortened resting length in the EHTs stimulated with ET-1 which clearly showed the strongest fibrotic changes. Resting length in the control, AE- or PE-EHTs was not different (Online Fig. IV).

### Functional consequences of afterload enhancement

**Fig. 7:**
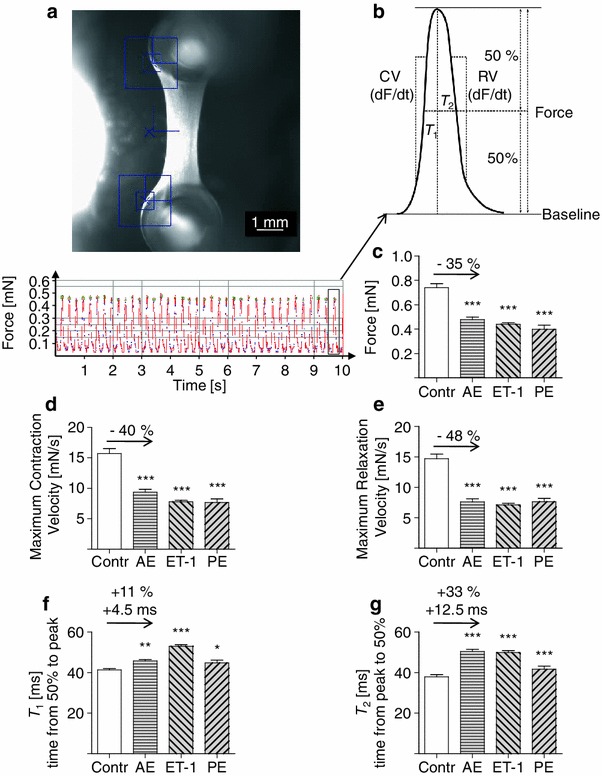
Functional consequences of hypertrophic interventions on EHTs. **a** Example of a video-optical recording of an EHT. *Blue* *squares* in the still image (*above*) indicate positions which are used to analyze EHT contractions. The corresponding contraction recording over a period of 10 s is depicted below. **b** Pictograph of one contraction peak and the values derived from it. Note that *T*_1_ and *T*_2_ are measured from or to the time of 50 % of maximum contraction. **c–g** Functional parameters of hypertrophied EHTs after removal of the metal braces in comparison to control EHTs. **c** Force of contraction. **d** Maximum contraction velocity. **e** Maximum relaxation velocity. **f** Contraction time *T*_1_. **g** Relaxation time *T*_2_. (all *n* = 8–9 per group)

Each EHT was filmed every other day and the contractions were recorded over time (Fig. 7a; Online Movie I). Important contraction parameters were determined from each peak and averaged by the CTMV software (Fig. 7b). All functional parameter of AE-EHTs were assessed 20 min after removal of metal braces and compared to control EHTs handled in parallel. Mean forces developed by control EHTs were 0.74 mN, but only 0.48 mN (−35 %) in the AE group, 0.44 mN (−41 %) in the ET-1-group, and 0.40 mN (−46 %) in the PE-group (Fig. 7c). The loss of contractile force was persistent over the follow-up period (Online Fig. V). For AE and ET-1 it was likely not due to a loss of total muscle mass, because the sum of the cross-sectional areas of dystrophin-stained myocytes (Fig. 3) did not differ between these groups and the control group (control: 0.17 mm^2^, AE: 0.17 mm^2^, ET-1: 0.19 mm^2^). As a result, normalized forces were similarly reduced than unnormalized forces in AE (−33 %), and ET-1 (−46 %). In the PE-group the above mentioned sum was 0.11 mm^2^, so myocyte loss might have contributed to the loss of contractile force in this group. Concomitantly, contraction (Fig. 7d) and relaxation velocities were lower in the hypertrophy groups, the latter being more pronounced (Fig. 7e). Since both parameters are partially dependent on force we also determined the largely force-independent parameters contraction time *T*_1_ (Fig. 7f) and relaxation time *T*_2_ (Fig. 7g). After AE, *T*_1_ was mildly (4.5 ms) and *T*_2_ was considerably longer (+12.5 ms, +33 %) than in control EHTs.

To distinguish whether impaired contractility reflected real effects of the hypertrophic interventions or simple injury of the tissue by mechanical manipulation, we inserted metal braces for only short periods of time. Even after repeating this procedure on three consecutive days it did not impair contractility, but rather had a slight stimulatory effect (Online Fig. VI). Markers of cell death or tissue injury remained unchanged compared to control EHTs. Interestingly, a comparison of EHTs cast on silicone posts with low spring constants (*k* = 0.29) to our standard spring constant without metal braces (*k* = 0.95) revealed higher forces and bigger cardiomyocytes indicating that at the low range of spring constants EHTs might also be suitable for investigating physiological hypertrophy (Online Fig. VI).

### Prevention of the deleterious afterload enhancement effects by endothelin receptor blockade

**Fig. 8:**
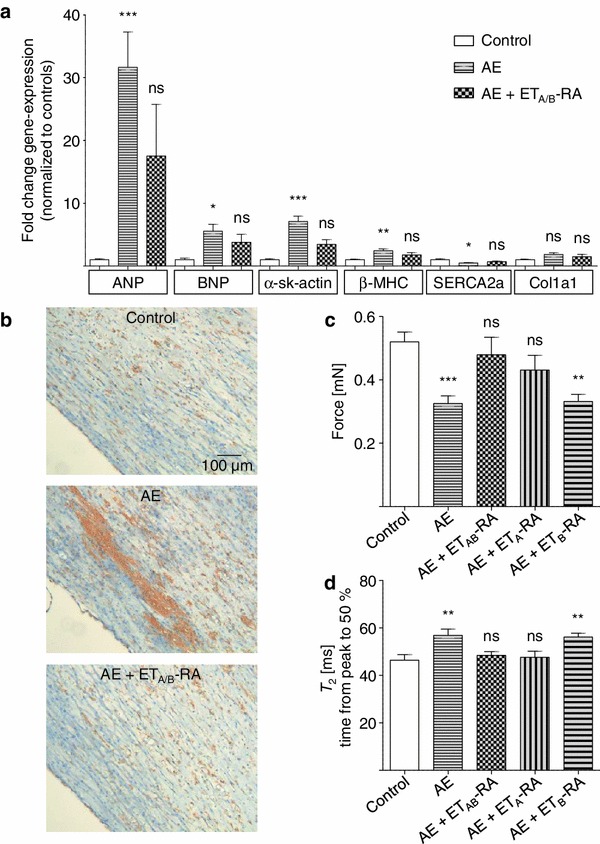
Prevention of the deleterious afterload enhancement effects by endothelin receptor blockade. **a** Transcript concentrations of members of the hypertrophic program as measured by RT-qPCR. Concentrations of AE-EHTs and AE-EHTs under blockade with the combined ET_A/B_-receptor antagonist (ET_A/B_-RA) PD 142893 were normalized to controls. (*n* = 5 biological × 3 technical replicates). **b** Collagen-1-immunohistochemistry as an indicator of fibrosis in control, AE-EHTs and EHT under ET_A/B_-receptor blockade. **c** Force and **d** relaxation time T_2_ of EHTs after removal of the metal braces in comparison to control EHTs. AE + ET_A/B_-RA were blocked with the combined ET_A/B_-RA PD 142893, whereas ET_A_-RA stands for selective ET_A_-blockade with BQ-123 and ET_B_ for selective ET_B_-blockade with BQ-788. (*n* = 10–15 per group)

The effects of AE and ET-1 on EHTs were strikingly similar with regard to gene expression cluster analysis (Fig. 4a), glucose metabolism, frequency or functional parameters. To investigate whether endothelin receptors participated in the mechanism of AE-induced remodeling, AE was induced in the presence of endothelin receptor antagonists (ET-RA). Activation of the hypertrophic gene program was partially blunted by ET_A/B_-RA (PD 142893, Fig. 8a). In addition, fibrotic changes in AE-EHTs were completely preventable by ET_A/B_-RA (Fig. 8b). The loss in contractile force (Fig. 8c) and the impaired relaxation (expressed as longer *T*_2_ time) following AE (Fig. 8d) was also mitigated by ET_A/B_-RA. The selective ET_A_–RA BQ-123 mimicked the effect of PD 142893, whereas the selective ET_B_-RA BQ-788 had no significant effect, suggesting the specific involvement of the ET_A_ receptor in AE-induced remodeling in EHTs.

## Discussion

In this study we developed a new model of cardiac hypertrophy based on our EHT technology. The main findings were as follows: (1) Low concentrations of triiodothyronine (*T*_3_) in cell culture medium allowed the cultivation of beating EHTs without serum for more than a week. (2) A sole increase of afterload of EHTs triggered the activation of the hypertrophic gene program and (3) lead to an increase in cardiomyocyte size, which was measurable by myocyte specific dystrophin staining. (4) Cardiomyocyte hypertrophy could also be induced by the α-agonist phenylephrine or endothelin-1. (5) Gene expression patterns in the mechanical and pharmacological groups showed large overlap, but were greatly different from control EHTs. (6) Glucose consumption per beat was increased in afterload enhanced EHTs. (7) All 3 hypertrophic interventions lead to fibrotic activation as well as sustained impairment of contractile force and relaxation both of which were preventable by endothelin receptor blockade. The data show, as we believe for the first time, that an increase in afterload, independent of systemic neurohumoral activation or a mismatch between blood supply and demand, is sufficient to induce the whole spectrum of pathological hypertrophy in cardiac muscle tissue.

Cardiac preload is defined as end-diastolic wall tension. In this study, we refer to preload as the tension exerted by the silicone posts on EHTs in the resting state (metered value 0.8 mN, calculated from the observed deflection of silicone posts in the resting state). The load opposing shortening of the ventricular muscle fibers is termed ventricular afterload [32]. This load in EHTs is the sum of the preload and the tension produced by the contraction of the EHT. The latter is considerably increased when EHTs have to beat against stiff metal braces. Besides neurohumoral activation chronically increased hemodynamic load is the main trigger of cardiac hypertrophy in patients [14]. We evaluated both in EHTs. On the one hand phenylephrine (α-adrenergic agonist) [17], and endothelin-1 (ET_A_ and ET_B_ receptor agonist) [26, 34] were chosen as well-established humoral inductors of cardiomyocyte hypertrophy. Both agonists bind to receptors which are coupled to G_q_/G_11_-proteins and subsequently activate phospholipase C. The consequence of mechanical load imposed on rat cardiomyocytes in vitro was evaluated in numerous studies by others [11] and us [10], or even on human cardiomyocytes derived from embryonic or induced pluripotent stem cells [39]. In these studies cardiomyocytes were stretched passively, i.e. they were subjected to preload enhancement [23]. Other in vitro models of cardiac hypertrophy on the basis of a short-term culture of excised rabbit trabeculae also investigated preload increases [5, 18]. However, data from large epidemiological studies such as the Framingham Heart Study revealed that afterload enhancement (and especially sustained hypertension) is far more important than preload enhancement for the development of heart failure [21]. This finding was also supported in a comparison of TAC mice (afterload) and shunt mice (preload) [37].

A crucial point in developing the new in vitro model of hypertrophy was to reduce hypertrophic culture medium supplements. Our previously published EHT protocol relied on culture medium containing 10 % horse serum [12]. As serum is a strong inducer of cardiomyocyte hypertrophy [35], we reduced horse serum concentration during EHT development and completely omitted it one day before starting hypertrophic interventions. This was possible by supplementing the medium with triiodothyronine (*T*_3_), well known to be essential for cardiac development [7, 31]. *T*_3_ itself is capable of provoking cardiomyocyte hypertrophy, although this form is usually referred to as physiological. Taking this into consideration, a *T*_3_ concentration in the lower physiological range was chosen, approximately 100-fold lower than that reported to induce hypertrophy [16].

The stimulation of EHTs with phenylephrine or endothelin-1 induced the canonical hypertrophic (or fetal) gene program with upregulation of ANP, BNP, β-MHC and α-skeletal actin as well as downregulation of SERCA2a [9, 38]. Remarkably, sole afterload enhancement showed almost an identical pattern not only regarding the direction but also the extent of changes. Importantly, altered transcript concentrations were accompanied by similar changes in the protein concentrations as shown for two representative examples (ANP and β-MHC). Of note, we chose the β-MHC antibody clone NOQ 7.5.4D, which has recently been reported by the Simpson group as the only specific available β-MHC antibody [22]. As nicely illustrated in that study, the fetal gene program does not necessarily indicate hypertrophy or even pathological hypertrophy. We therefore concentrated on precisely determining cardiomyocyte size. Cell sizes were measured in situ, i.e. in fixed native EHTs, to prevent any changes in cell morphology caused by enzymatic isolation from their extracellular matrix. A specific sarcolemma staining with a dystrophin antibody ensured that only myocytes were included in the analysis and facilitated automated and blinded measurement of cardiomyocyte sizes. The increase in cross-sectional area of cardiomyocytes by approximately 30 % was similar in all hypertrophy groups and thus within a reasonable and frequently reported range [1, 15].

The microarray analysis displayed that control and hypertrophied EHTs differed distinctly in their expression patterns. The upregulated genes in AE-EHTs displayed modest overlap with upregulated genes in mouse TAC. However, even gene expression between two identical procedures in the same species (Mouse TAC I and II) was widely different. Strikingly, the total overlap of upregulated genes in all three groups (with short-term TAC II) was very consistent with the “hypertrophic gene program” and the overlap increased considerably (particularly by fibrotic markers) when including long-term TAC II, which the authors referred to as failing phenotype [20].

Two KEGG pathways, *Glycolysis* and *Extracellular Matrix*, were identified in the enrichment analysis of the transcriptome to be overrepresented in the panel of upregulated genes of all three hypertrophy groups. Alterations in cardiomyocyte energetics in pathological cardiac hypertrophy with a shift from fatty acids (normally 60–90 %) to glucose as the primary myocardial energy substrate are described [3, 8, 27, 36]. However, these experiments relied on hypertrophied myocardium (in vivo*)* which after explantation was analyzed in vitro. We observed an upregulation of glycolytic genes and an increase in glucose consumption per beat (+48 %) in EHTs hypertrophied by AE—despite lower contractile forces. To our knowledge, this is the first time this metabolic shift has been observed in vitro in its entirety. Apparently, cardiac tissue has a native metabolic plasticity without the need for regulation by the whole organism.

Cardiac fibrosis is an undisputed criterion of pathological hypertrophy [6]. Increased collagen-1 deposition has been associated with increased myocardial stiffness [2, 28] and a decrease in the elastin to collagen ratio has been found in decompensated cardiac hypertrophy [25]. In our model, the increased gene expression of several pro-fibrotic genes, the downregulation of fibrillin and elastin and the immunohistochemical detection of increased deposition of freshly synthesized collagen-1 indicate that afterload enhancement as well as the pharmacological interventions induced a fibrotic response as part of a pathological remodeling process. These findings highlight two advantages of EHTs. On the one hand, fibrin as ECM for tissue engineering allows unambiguous detection of freshly synthesized collagen-1 whereas collagen-1 as used by us previously [43] does not. On the other hand, EHTs likely enable “more physiological” crosstalk between cell types than 2-D cultures, because all native ventricular heart cell types are present and organized in a 3-D tissue-like structure. The present findings indeed show that EHTs allow studying processes as complex as cardiac fibrosis, known to involve activated fibroblasts (myofibroblasts), endothelial cells, cardiomyocytes and monocytes [2, 24]. Whether the remodeling involved (cell-type-specific) changes in cell proliferation was not evaluated and requires further studies.

A unique advantage of EHTs is the possibility to measure contractile function over a long period of time. The observed 35 % loss of contractile force after AE was initially surprising because it is to be viewed on the background of increased myocyte size (+28 %). However, the sum of myocyte cross-sectional area in AE was almost identical to that in control, indicating loss of some myocytes on the one hand and reduced force generation per muscle mass on the other. Moreover, relaxation of AE-EHTs was clearly impaired as it is known from hypertrophied myocardium [40]. These parameters suggest that the cardiomyocyte hypertrophy after AE was pathological and not the consequence of a physiological training effect (which we observed when we increased spring constants of the elastic posts from *k* = 0.29 [solid] to 0.95 [hollow]) or reversal of an unphysiological “starving process” due to lack of serum. An unambiguous classification of cardiac hypertrophy into a physiological or pathological form is usually not possible due to intermediate conditions [8]. However, most phenomena we observed are generally attributed to pathological hypertrophy: increase of α-skeletal actin and β-MHC with concomitant decrease of thyroid hormone receptors α and β [16], decrease of SERCA2a, a metabolic shift towards glycolysis, G_q_-coupled signaling, cardiac fibrosis, loss of contractility, and impaired relaxation [8, 14]. We believe that these observations are important for several reasons. First, as far as we are aware of, it is the first time that all of these hallmarks of pathological hypertrophy can be measured and are reproduced together in an in vitro model. Second, it shows that afterload enhancement as such induces pathological hypertrophy, independent of other well known factors such as systemic neurohumoral activation [13] and the mismatch between the growth of blood vessels and myocytes [33]. Intriguingly, the deleterious effects of afterload enhancement on contractile parameters and fibrosis were preventable by endothelin blockers, nicely supporting either the early concept of autocrine or paracrine release of ET-1 and Ang II [30, 42], or a direct mechanically induced activation of ET_A_-receptors as proposed for angiotensin II-receptors [44], or both. The fact that we did not find altered endothelin gene expression (data not shown) argues for the predominant importance of the ligand-independent mechanism. Third, the model is simple, robust and, in contrast to other cardiomyocyte cell culture techniques, stable for weeks. It is highly standardized, reproducible and can be easily established in any standard laboratory with animal facility. Thus, it will be useful in investigating molecular processes involved in pathological cardiac hypertrophy and in evaluating potential therapeutic approaches.

## Electronic supplementary material

Below is the link to the electronic supplementary material.

Supplementary material 1 (PDF 777 kb)

Supplementary material 2 (AVI 1287 kb)

Supplementary material 3 (AVI 1434 kb)

## Abbreviations

AE: Afterload enhanced or enhancement
CV: Contraction velocity
ECM: Extracellular matrix
EHT: Engineered heart tissue
ET-1: Endothelin-1
ET_A/B_-RA: Nonselective endothelin receptor antagonist
ET_A_-RA (ET_B_-RA): Selective endothelin-A receptor antagonist (endothelin-B)
PE: Phenylephrine
RV: Relaxation velocity
*T*_1_: Contraction time
*T*_2_: Relaxation time
*T*_3_: Triiodothyronine
TAC: Transverse aortic constriction
