# Supplementary material for: Increased afterload induces pathological cardiac hypertrophy: a new in vitro model
Source: Basic Res Cardiol. 2012 Oct 26;107(6):307. doi: 10.1007/s00395-012-0307-z (PMC3505530; doi:10.1007/s00395-012-0307-z)
Supplement: Supplementary file 1 — Supplementary material 1 (PDF 777 kb) [file 395_2012_307_MOESM1_ESM.pdf]

SUPPLEMENTAL MATERIAL

Increased afterload induces pathological cardiac hypertrophy: a new in vitro model

Marc N. Hirt*[1], Nils A. Sörensen*[1], Lena M. Bartholdt[1], Jasper Boeddinghaus[1], Sebastian Schaaf[1], Alexandra Eder[1], Ingra Vollert[1], Andrea Stöhr[1], Thomas Schulze[1], Anika Witten[2], Monika Stoll[2], Arne Hansen[1], Thomas Eschenhagen[1]

[1]Department of Experimental Pharmacology and Toxicology, University Medical Center Hamburg-Eppendorf, and DZHK (German Centre for Cardiovascular Research), partner site Hamburg/Kiel/Lübeck, Germany, [2]Leibniz Institute for Arteriosclerosis Research, Münster, Germany
* equal contribution

Materials and Methods

RNA isolation

RNeasy® (Qiagen) was utilized to prepare total RNA from EHTs. Only the tissue between the two silicone tubes, which potentially had the highest workload, was used for it (and not the tissue which grew around the silicone discs, Fig. 1e). Apart from an antecedent Proteinase K incubation to digest fibrin, manufacturer's instructions were followed. RNA-concentration and purity were determined with a Nanodrop*TM* ND-1000 (Thermo Fisher Scientific Inc.) photometer.

Quantification of gene expression

For reverse transcription and quantitative PCR High Capacity cDNA Reverse Transcription Kit (Applied Biosystems) and POWER SYBR® Green PCR Master Mix (Applied Biosystems) were used according to manufacturer's instructions. Experiments were performed on ABI PRISM 7900HT Sequence detection system (Applied Biosystems). Multiple internal control genes were used to countervail variability. Primer sequences are listed in Online Table VI.

Histology

The middle sections of EHTs (Fig. 1e) were fixed with formaldehyde both for conventional hematoxylin and eosin (H&E) staining and immunohistochemistry. After embedding in paraffin 4 µm sections were cut strictly longitudinally or transversally. H&E staining was performed according to standard protocols in an automated manner. For immunohistochemistry, staining conditions for each antibody were optimized on paraffin sections of adult rat hearts. 30 different combinations of antigen retrieval and antibody dilution were tested on the Ventana® BenchMark® XT (Roche) slide preparation system. Optimized conditions were: mouse anti-dystrophin monoclonal antibody (Millipore, MAB1645), dilution 1:200, antigen retrieval: 60 min in EDTA-buffer, pH 8.0; mouse anti-ANP (23/1) monoclonal antibody (Santa Cruz, sc-80686), dilution 1:500, antigen retrieval: 60 min in citrate-buffer, pH 6.0; rabbit anti-collagen 1 polyclonal antibody (Abcam, ab34710), dilution 1:200, antigen retrieval: 60 min in citrate-buffer, pH 6.0. All antibodies were visualized with the multimer-technology based UltraView Universal DAB Detection Kit (Roche). All microscopic images were taken on an Axioskop 2 microscope (Zeiss).

Cell size analysis

Cross sections of EHTs were stained for dystrophin and analyzed with the AxioVision Rel. 4.8 software (Zeiss). Cross-sectional area of each dystrophin positive cell was determined with the AutoMeasure module using automatic segmentation. The investigator was blinded to allow unbiased minor manual refinements of segmentation. The minimum threshold for cross-sectional area was set at 40 μm^2, and 150 dystrophin positive cells were analyzed per group. The sum of all cross-sectional areas (sum CSA) of dystrophin-stained myocytes was used for normalization of contractile forces to myocardial mass (force / sum CSA) with the limitation that numerator and denominator were obtained from two different experimental series.

Immunofluorescence

For confocal laser scanning microscopy, EHTs were processed as whole mounts. Each step, formaldehyde-fixation, blocking and permeabilization, incubation with primary antibodies and incubation with secondary antibodies, was performed for 24 hours at 4°C. Confocal images were obtained with a Zeiss LSM 510 META System. Antibodies were used as follows: α-actinin 1:800 (Sigma-Aldrich A7811), Alexa Fluor® 488 (goat anti-mouse IgG 2 mg/mL, Invitrogen A11001). Nuclei were counterstained with DRAQ5 1:1000 (Biostatus Limited DR50050). Block Solution: TBS 0.05 M, pH 7.4, 10 % FCS, 1 % BSA, 0.5 % Triton X-100; antibody solution: TBS 0.05 M, pH 7.4, 1 % BSA, 0.5 % Triton X-100.

SDS-Page/Western-Blot analysis

The middle sections of EHTs (Fig. 1e) were homogenized in lysis buffer (Tris 30 mM at pH 8.8, 3 % SDS, 10 % glycerol, EDTA 5 mM, sodium fluoride 30 mM, and aprotinin 2 mg/L) in a mechanical sample disruption system with stainless steel beads (Qiagen Tissue Lyser). For immunoblotting, aliquots of denatured protein were subjected to SDS-PAGE on a 10 % polyacrylamide gel, and separated proteins were electrophoretically transferred onto nitrocellulose membranes. Nonspecific binding was blocked by incubation with 5 % nonfat dry milk for 2 h at room temperature. Membranes were then incubated with the NOQ7.5.4D anti-β-MHC monoclonal antibody (1:2,000; Sigma-Aldrich M8421) or anti-p44/42 MAPK (Erk1/2) polyclonal antibody (1:1,000, Cell Signaling #9102) overnight at 4°C. After being washed in TBS buffer containing 1 % Tween 20, the membranes were incubated with horseradish peroxidase-conjugated secondary antibodies (1:20,000 and 1:5,000, respectively) for 1 h at room temperature. The immunoreactive bands were visualized by ECL detection (Pierce #32106).

Whole transcriptome analysis

RNA was isolated (see above) from 6 EHTs per group (control, AE, ET-1, PE). According to manufacturer's protocol 250 ng RNA was subjected to first and second strand synthesis, followed by

an in vitro transcription (IVT) amplification that incorporated dU-biotin (Illumina TotalPrep Amplification Kit, Ambion). Subsequently cRNA was purified and hybridized on RatRef-12 Expression BeadChips containing 21,910 probes (Illumina BD-27-302). Arrays were washed, blocked, and streptavidin-Cy3 stained for reading on a BeadArray Reader (Illumina). Gene expression levels and sample clustering by correlation were calculated after quantile normalization using GenomeStudio version 1.6.0 software (Illumina). Differentially expressed genes were identified using the Illumina custom error model, multiple testing correction was performed using Benjamini and Hochberg False Discovery Rate. Data-mining was performed with DAVID Bioinformatics Resource 6.7 (NIAID, NIH) according to a recently published protocol [3]. Its functional annotation chart module was utilized to investigate whether KEGG pathways (Kyoto Encyclopedia of Genes and Genomes) were overrepresented in our differentially expressed genes [4].

Up- or downregulated genes in the rat afterload-enhanced EHTs were compared with published gene lists of transverse aortic constriction in mice. Rat gene symbols were converted to mouse gene symbols using the Mouse Genome Database (MGD) from the Mouse Genome Informatics website, The Jackson Laboratory, Bar Harbor, Maine (www.informatics.jax.org; 08/0212) [1]. The mouse TAC I data set was taken from the supplemental data of an article from Toischer et al. [6]. The mouse TAC II data set from Lee et al. [5] was obtained via the Gene Expression Omnibus of the NCBI (accession: GSE 29446). Whenever applicable lists were cured with the DAVID Bioinformatics Resource 6.7.

Glucose consumption

The glucose content of the EHT cell culture medium was measured with a blood gas analyzer (ABL90 FLEX, Radiometer GmbH). Glucose consumption (in 24 hours) of beating EHTs was calculated by subtracting the measured glucose concentration after 24 hours from the known glucose concentration in fresh medium and subsequent multiplying by the medium volume (1.5 mL).

Macroscopic dimensions of EHTs

The CTMV software (Pforzheim, Germany) captured pictures of each analyzed EHT automatically. Diameters of EHTs were measured from these images at the narrowest point in diastole with the ImageJ 1.44p software (National Institute of Health). Resting lengths of EHTs in diastole were measured identically.

Electron microscopy

EHTs were incubated in 2,3-butanedione monoxime (BDM, Sigma-Aldrich B0753) at 30 mM for 10 minutes to stop cardiac muscle contraction and allow uniform relaxation. After overnight fixation in 0.36 % glutardialdehyde EHTs were postfixed with 1 % osmium tetroxide for 2 h. Dehydration of samples was followed by embedding in epon. Ultrathin sections of 50 nm were prepared, contrasted with uranyl acetate and lead nitrate and examined under a LEO 912AB transmission electron microscope with Omega energy filter (formerly from Zeiss).

Hormone measurements and determination of enzyme activity

Free and total triiodothyronine (T_3) concentrations as well as lactate dehydrogenase (LDH) and creatine kinase (CK) enzyme activity were measured in appropriate medium without phenol red. All analyses were performed with routine tests in a Clinical Chemistry Department.

Statistics

Results are presented as mean±SEM. All statistical tests were performed in GraphPad Prism version 5.02. In detail, 1-way ANOVA and Dunnett‘s multiple comparison post test (to compare to controls) or 1-way ANOVA and Bonferroni’s multiple comparison post test (to compare all groups) was used for more than 2 groups, or Student’s unpaired t test for 2 groups. Repeated measures 1-way ANOVA followed by Dunnett‘s multiple comparison post test (to compare to controls) was performed for matched measurements (Online Fig. V). Non-Gaussian data was analyzed by Kruskal-Wallis test and Dunn’s multiple comparison post test. $P<0.05$ or less was considered statistically significant. P-values

are displayed graphically as follows: * $p<0.05$, ** $p<0.01$, *** $p<0.001$, ns = not significant. Quantitative PCR data analyses were carried out using the ΔΔ-Ct method.

Online Figures, Tables and legends

**Reconstitution mix to generate 24 EHTs**	
**Rat ventricular heart cells**	**16.7 x 10^6**
**DMEM (2x)***	**186.7 µL**
**Bovine fibrinogen****	**86.7 µL**
**Medium 1*****	**ad 3333 µL**

Stock solutions:

* DMEM (2x): 20 % DMEM (10x), 20 % horse serum inactivated (Gibco 26050), 2 % penicillin/streptomycin (Gibco 15140), 4 % chick embryo extract, all in water

** Bovine fibrinogen: fibrinogen (Sigma-Aldrich F4753) 200 mg/mL + aprotinin (Sigma-Aldrich A1153) 0.5 µg per mg fibrinogen, all dissolved in NaCl 0.9 %

*** Medium 1: 10 % fetal calf serum inactivated (Gibco 26010), 1 % penicillin/streptomycin (Gibco 15140), 1 % glutamine (Gibco 25030), all in DMEM (Biochrom F0415)

Online Table I

Composition of the reconstitution mix to generate EHTs. Reconstitution mix was prepared on ice and blended carefully. For each EHT 100 µL of it was mixed briefly with 3 µL thrombin (100 U/mL, Sigma-Aldrich T7513) and pipetted into the agarose slot, already containing the tubes of the silicone rack

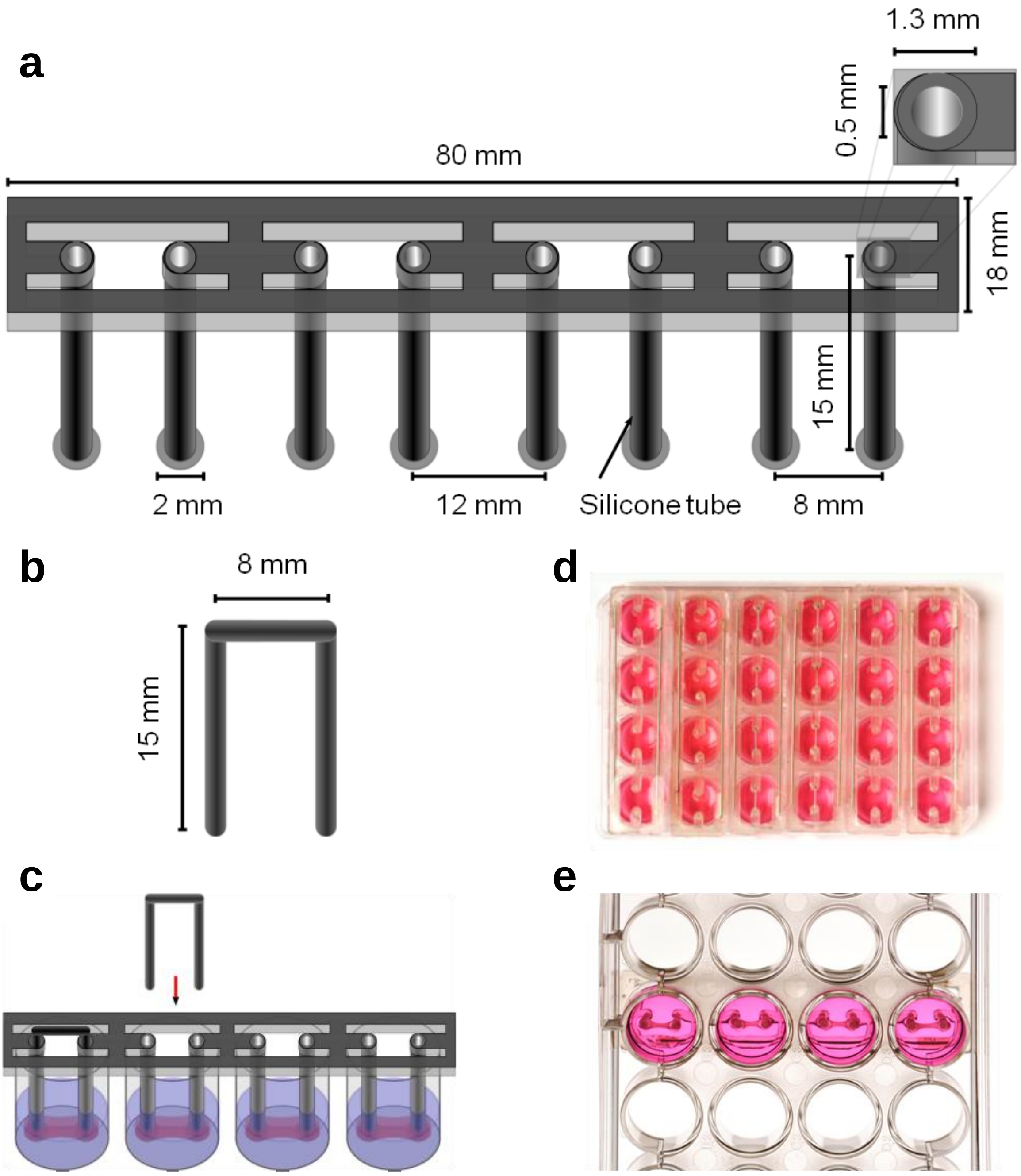

Online Fig. I

Illustration and photographs of materials used for the model of afterload enhancement in EHTs. **(a)** Illustration of a silicone rack carrying 4 pairs of silicone tubes fitting into a standard 24-well cell culture dish. **(b)** Stainless steel metal braces. **(c)** Illustration of the experimental procedure. Metal braces were inserted into the silicone tubes. **(d)** Complete 24-well culture dish. The middle 8 EHTs were afterload enhanced with metal braces. **(e)** 4 EHTs in a bottom-up perspective

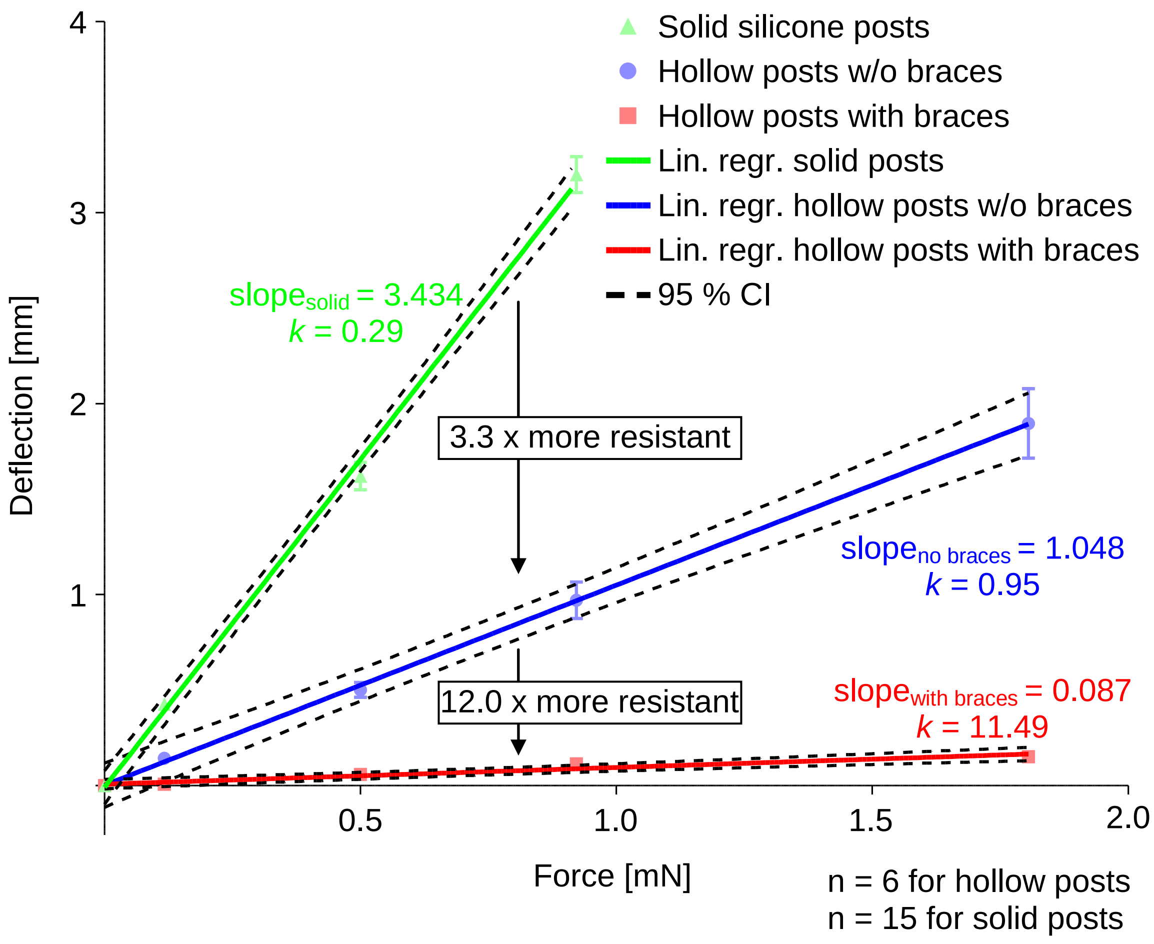

Online Fig. II

Determination of spring constants *k* for the silicone posts without metal braces (red, i.e. empty silicone tubes) and the same silicone posts with inserted metal braces (blue). Deflection in millimeters was measured as a function of force subjected to the silicone posts. Measured values are depicted in pale colors (mean ± SEM), corresponding linear regression curves in dark colors, and the 95 % confidence band as black dotted lines. Spring constant *k* for silicone tubes without metal braces (subsequently referred to as controls) was 0.95 N/m, after insertion of metal braces (referred to as afterload enhanced) the spring constant rose by a factor of 12 to 11.5. (*k* is the reciprocal value of the slope.) Green values and line derive from solid silicone tubes used in previous studies [2]. They were crafted from the same material; their lower resistance was solely due to being solid instead of hollow

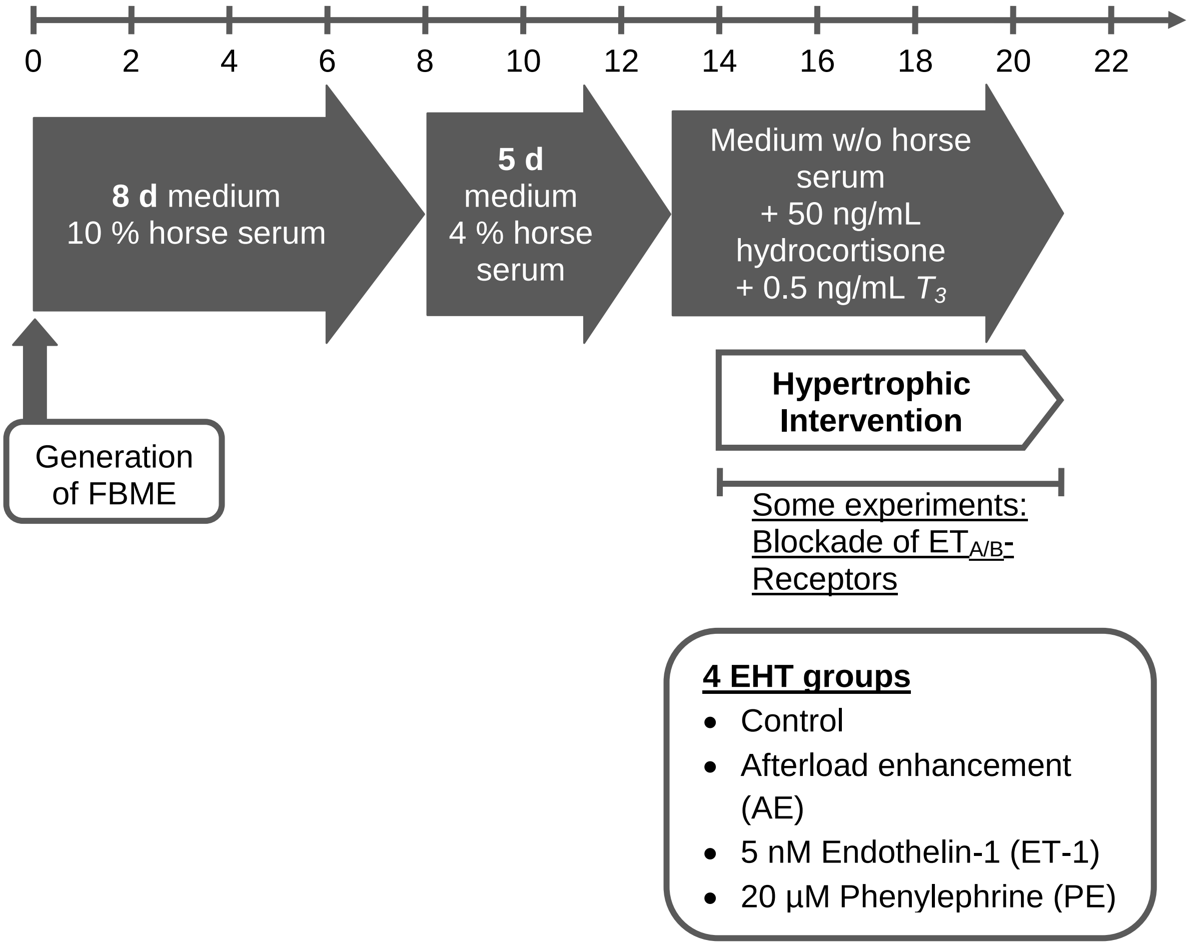

Online Fig. III

Standard timeline for the in vitro model of cardiac hypertrophy

**Endothelin Receptor Antagonist**	**Name**	**Source/ Catalog- Nr**	**IC_{50}-ET_A**	**IC_{50}-ET_B**	**Used Concentration**
**Nonselective antagonist ($ET_{A/B}$-RA)**	PD 142893	Sigma/ #SCP0134	58 nM	130 nM	**10 µM**
**Selective ET_A-antagonist (ET_A-RA)**	BQ-123	Sigma/ #B150	70 nM	18µM	**1 µM**
**Selective ET_B-antagonist (ET_B-RA)**	BQ-788	Sigma/ #B157	1300 nM	30 nM	**300 nM**

Online Table II

Endothelin receptor antagonists (ET-RA) used in this study

	Conventional EHT medium (10 % horse serum)	T_3-supplemented, horse serum-free medium (0 % horse serum + 0.5 ng/mL T_3)	Reference range (human)
**Free T_3**	0.65 pg/mL	0.4 pg/mL	2.2 – 5.5 pg/mL
**Total T_3**	0.7 ng/mL	0.7 ng/mL	0.8 – 2.0 ng/mL

Online Table III

Total and free T_3-concentrations measured in conventional and T_3-supplemented, horse serum-free medium. The 0.2 ng/mL deviation of total T_3-concentration in the T_3-supplemented medium (measured vs. added) was likely due to the presence of chick embryo extract 2 %

AE			ET-1			PE		
**Rank**	**Gene**	**FC**	**Rank**	**Gene**	**FC**	**Rank**	**Gene**	**FC**
1	*Scn3b*	4,55	1	*Pap*	8,54	1	*Pap*	10,95
2	***Myh7***	3,96	2	*Ak3l1*	6,09	2	*Slc16a3*	10,37
3	***Acta1***	3,62	3	*Slc16a3*	5,9	3	*Hamp*	9,74
4	*Slc16a3*	3,32	4	*Thbs4*	4,51	4	*Ak3l1*	9,6
5	***Nppa***	3,31	5	*Bnip3-ps1*	4,44	5	*Bnip3-ps1*	7,73
6	*S100g*	3,25	6	*Egln3*	4,38	6	*Cdh17*	7,03
7	*Scd1*	3,18	7	*A2m*	4,37	7	*Scd1*	6,76
8	*Enah*	3,05	9	*Hamp*	4,15	8	*Bnip3*	6,75
9	*Usmg5*	3,01	8	*Bnip3*	4,15	9	*Eno2*	6,59
10	*Tbx15*	2,96	10	*LOC312273*	4,11	10	*Ankrd37*	6,42
11	*Arhgap11a*	2,87	11	*Maoa*	4,02	11	*Fstl3*	6,05
12	*A2m*	2,81	12	*Aldoc*	3,99	12	*C1qr1*	5,36
13	*S100a4*	2,79	13	*Eno2*	3,81	13	*Mt1a*	5,28
14	*Ak3l1*	2,77	14	*Scd1*	3,53	14	*Maoa*	5,22
15	*Nuf2*	2,76	15	*Ankrd37*	3,48	15	*Aldoc*	5,16
16	*Grem1*	2,75	16	***Nppa***	3,24	16	*Ptprr*	5,11
17	*Prc1*	2,73	17	*Serpine1*	3,11	17	*Gbe1*	4,86
18	*Rpl39*	2,72	18	*Fstl3*	3,1	18	*Bhlhb2*	4,55
19	*Acat2*	2,67	19	*Pnoc*	3,09	19	*Lmcd1*	4,45
20	*Cks2*	2,66	20	*Ptgs2*	3,06	20	*Pygl*	4,42
21	*Mybpc2*	2,64	21	*Lmcd1*	2,96	21	*Egln3*	4,33
22	*Otud1*	2,6	22	*Serpine1*	2,88	22	*A2m*	4,26
23	*Bub1*	2,59	23	*Oldlr1*	2,88	23	*Pla2g7*	4,24
24	*Pbk*	2,57	24	*Uap1*	2,84	24	*Pdk1*	4,18
25	*Xirp2*	2,53	25	*Grem1*	2,83	25	*Ero1l*	4,12
26	*Vldlr*	2,52	26	*S100a4*	2,82	26	*Kcnk1*	4,08
27	*Bnip3-ps1*	2,51	27	*Pygl*	2,74	27	*LOC498829*	4,07
28	*Med21*	2,48	28	*Ptpro*	2,74	28	***Nppa***	4,06
29	*Bnip3*	2,47	29	*Uap1l2*	2,68	29	*RGD156490*	4,03
30	*Ptgs1*	2,47	30	*S100g*	2,67	30	*Ltb4dh*	3,93
60	***Nppb***	2,17	46	***Myh7***	2,33	54	***Acta1***	3,01
			96	***Nppb***	1,96	119	***Myh7***	2,41
			233	***Acta1***	1,64	128	***Nppb***	2,35

Online Table IV

The 30 most strongly upregulated genes in the 3 hypertrophy groups. Fold change is calculated to controls. Members of the canonical hypertrophic gene program are highlighted (bold). *Nppa* encodes for ANP, *Nppb* for BNP, *Acta1* for α-skeletal actin, *Myh7* for β-MHC. (FC = fold change to controls)

Gene symbol	Full name	Gene ID	Fold change (AE vs. ctrl)	Fold change (ET-1 vs. ctrl)	Fold change (PE vs. ctrl)
**Components of cardiac extracellular matrix**					
Col1a1	collagen, type I, alpha 1	29393	1.55 ***	1.81 ***	1.33 **
Col3a1	collagen, type III, alpha 1	84032	(1.27 ns)	1.25 *	0.68 ***
Fn1	fibronectin 1	25661	1.48 ***	2.16 ***	2.08 ***
Eln	elastin	25043	0.40 ***	0.51*	0.12 ***
Fbn1	fibrillin-1	83727	0.55 ***	0.64 ***	0.51 ***
Fbn2	fibrillin-2	689008	(0.93 ns)	(0.89 ns)	0.68 ***
**Profibrotic stimuli**					
Tgfb1	transforming growth factor, beta 1	59086	(0.79 ns)	(1.06 ns)	(1.17 ns)
Ctgf	connective tissue growth factor	64032	1.92 ***	1.86 ***	1.70 ***

Online Table V

Transcript concentrations of important structural components of cardiac extracellular matrix (ECM) and of regulatory proteins which promote fibrosis. (All fold changes are normalized to control EHTs, ns = not significant, * $p<0.05$, ** $p<0.01$, *** $p<0.001$, all n = 6 per group)

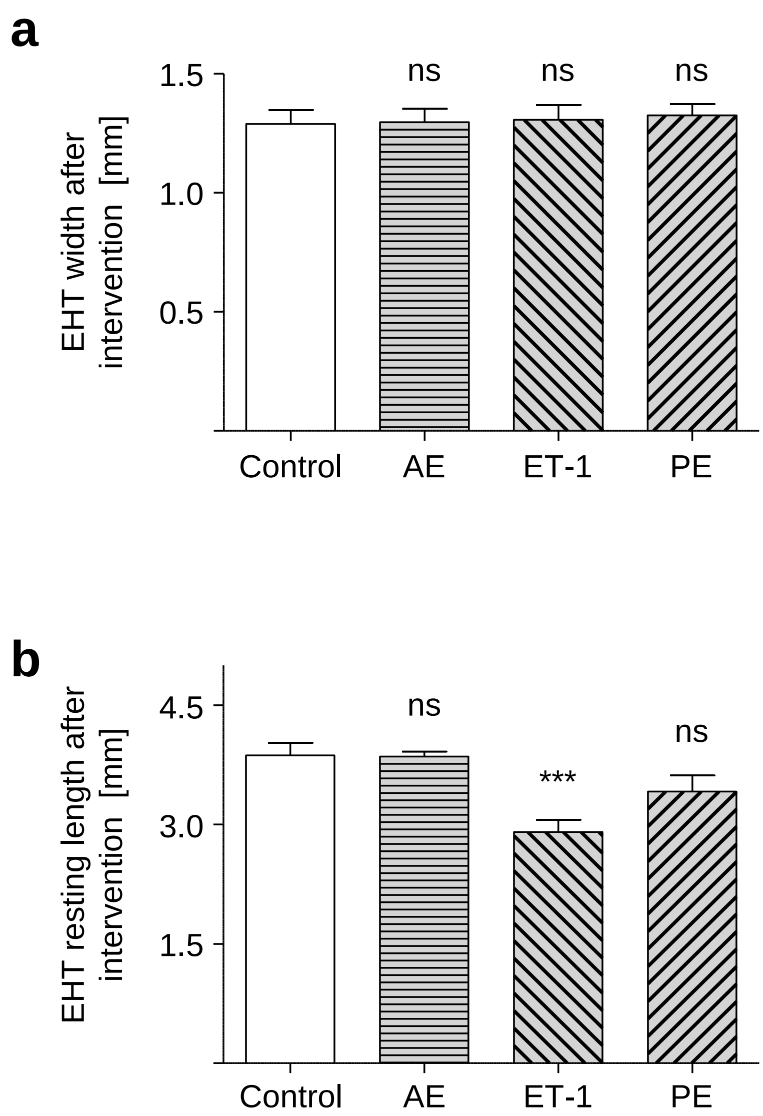

Online Fig. IV

(a) Diastolic EHT width at the narrowest point after hypertrophic interventions. The average diameter of EHTs was 1.3 mm in all groups. (n = 10 – 11 each). **(b)** Diastolic length, measured from post to post after hypertrophic interventions. Apart from the ET-1 group, which showed a decrease in resting length, there was no change in diastolic length. (n = 8 – 9 each)

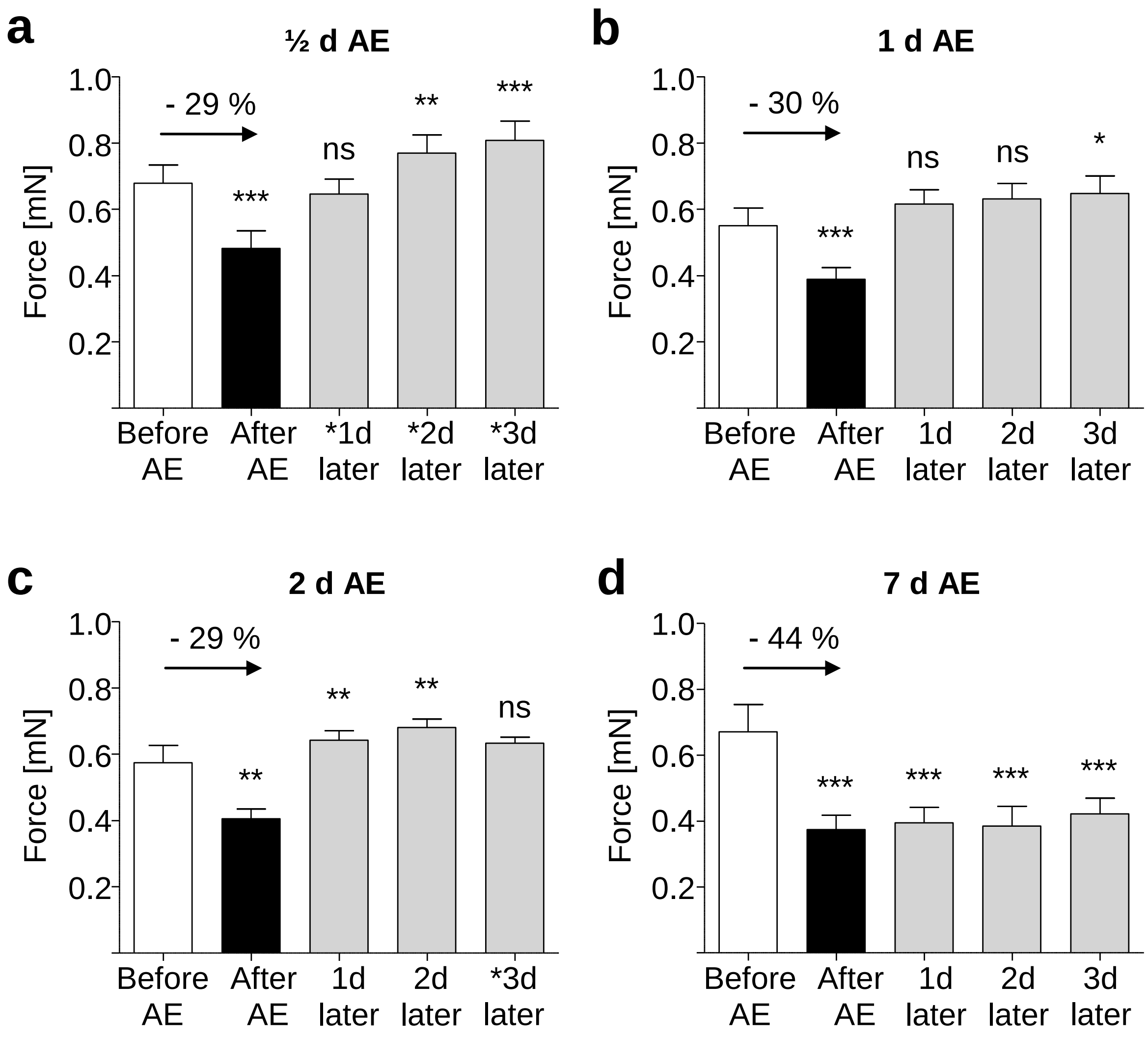

Online Fig. V

Comparison between short-term afterload enhancement (AE) ranging from **(a-c)** ½ day to 2 days with our standard long-term **(d)** 7 days afterload enhancement. Long-term AE lead to a greater loss of contractile force directly after intervention which persisted over the follow-up period of 3 days. Values denoted with asterisks (*) were calculated/interpolated as the mean values from a time point 12 hours earlier and a time point 12 hours later. (n = 5 - 8 per group)

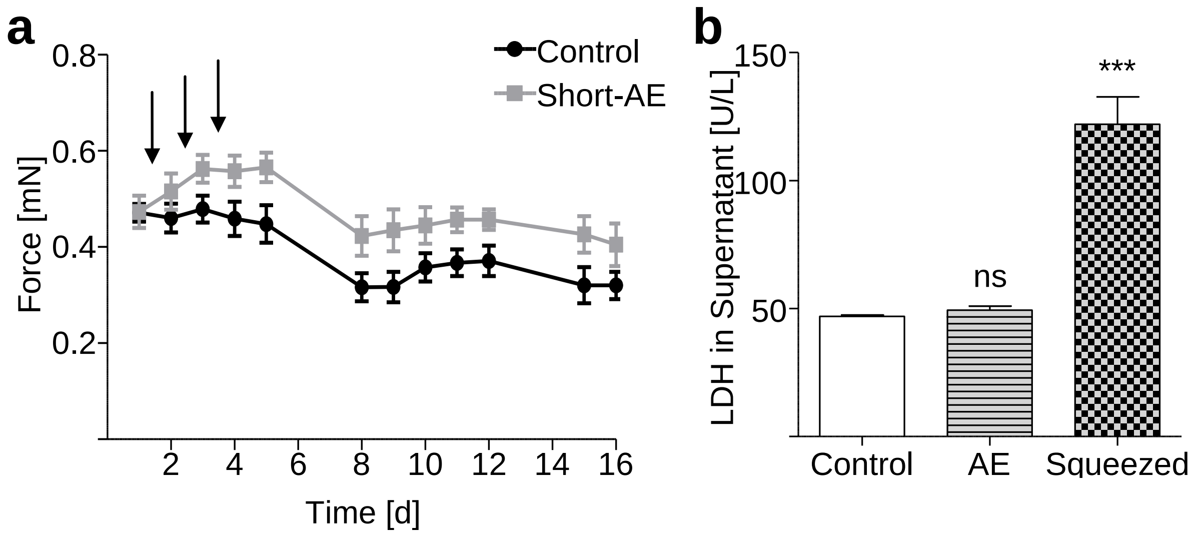

c

	Creatine Kinase in Supernatant
Control (n = 6)	**not detectable**
AE (n = 6)	**not detectable**
Squeezed (n = 3)	**+**

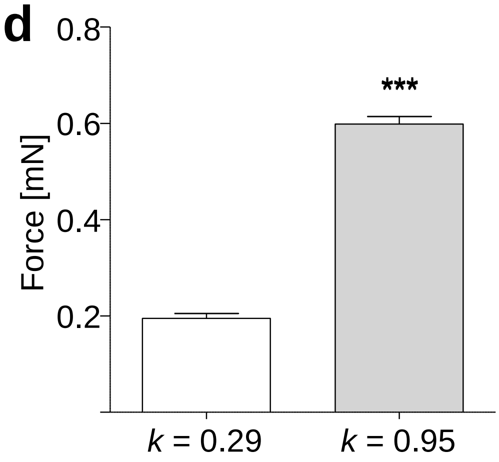

Online Fig. VI

(a) Short-term afterload enhancement (braces were inserted for 20 seconds; marked by arrows) on three consecutive days did not lead to a force decline, but a slight increase. (n = 6 per group). **(b)** On day 4 lactate dehydrogenase (LDH) as marker of cell injury was measured in supernatant of control, afterload enhanced and EHTs squeezed with a forceps which served as positive controls for cell injury. There was no difference between control and afterload enhanced EHTs. (n = 6 per group). **(c)** Creatine kinase (CK), a marker for myocyte injury, was detectable if the concentration in the supernatant exceeded 7 U/L. It was only detectable in the squeezed group. **(d)** Comparison of maximum forces of EHTs cast on solid silicone posts (k = 0.29) and on hollow silicone posts (k = 0.95, Online Fig. II). EHTs on hollow posts developed higher maximum forces. (n = 10 – 11 per group)

Gene symbol	Full name	Nm number	primer forward	primer reverse	PCR product size
Nppa	atrial natriuretic factor	NM_012612.2	CCTCGGAGCCTGCGAAGGTCA	TGTGACACACCGCAAGGGCTTG	156
Nppb	brain natriuretic peptide	NM_031545.1	GACGGGCTGAGGTTGTTTTA	ACTGTGGCAAGTTTGTGCTG	195
Acta1	α-skeletal actin	NM_019212.2	AGGACCTGTACGCCAACAAC	ACATCTGCTGGAAGGTGGAC	195
Myh7	myosin, heavy chain 7, cardiac muscle, beta	NM_017240.1	AGGAGGCGGAGGAACAGGCCAAC	GGGCTTCACAGGCATCCTTAGGGTT	200
Myh6	myosin, heavy chain 6, cardiac muscle, alpha	NM_017239.2	GGGGCAAGGTCACTGCCGAA	GGCCGCATAGCGCTCCTTGA	167
Atp2a2	SERCA2a	NM_001110139.2	TGCTGGAACTTGTGATCGAG	AGCGTTTCTCTCCTGCCATA	191
Col1a1	collagen, type I, alpha 1	NM_053304.1	TGGACCTCCGGCTCCTGCTC	TCGCACACAGCCGTGCCATT	172
Col3a1	collagen, type III, alpha 1	NM_032085.1	CTGTCCCGCGGAAGCACTGG	ATGTTCTGGGAGGCCCGGCT	178
Ctgf	connective tissue growth factor	NM_022266.2	GCGAGCCAACTGCCTGGTCC	GCGTCCGGATGCACTTTTTGCC	194
Fn1	fibronectin 1	NM_019143.2	CGGTGGCATGGAGAGCCAGC	TCACCCGCACTCGGTAGCCA	152
Gapdh	glyceraldehyde-3-phosphate dehydrogenase	NM_017008.3	CTCATGACCACAGTCCATGC	TTCAGCTCTGGGATGACCTT	155
18s	18s rRNA	V01270.1	ATACATGCCGACGGGCGCTG	TTCGAATGGGTCGTCGCCGC	181
Tfrc	transferrin receptor	NM_022712.1	TGCTTGGGTAGGAGGCAGCG	GCCGAGCAAGGCTAAACCGGG	186
Gusb	glucuronidase	NM_017015.1	CCCGCATGTCCCCAAGACGG	CGGCACGGAAGCTCCACAGG	153

Online Table VI

Primers used for quantitative PCR. Whenever applicable one primer of the primer pair spanned an exon-exon junction and primers were separated by at least one intron. All primers were designed for *rattus norvegicus*

Supplemental Reference

1. Eppig JT, Blake JA, Bult CJ, Kadin JA, Richardson JE (2012) The Mouse Genome Database (MGD): comprehensive resource for genetics and genomics of the laboratory mouse. Nucleic Acids Res 40:D881-886. doi:10.1093/nar/gkr974

2. Hansen A, Eder A, Bonstrup M, Flato M, Mewe M, Schaaf S, Aksehirlioglu B, Schworer A, Uebeler J, Eschenhagen T (2010) Development of a drug screening platform based on engineered heart tissue. Circ Res 107:35-44. doi:10.1161/CIRCRESAHA.109.211458

3. Huang da W, Sherman BT, Lempicki RA (2009) Systematic and integrative analysis of large gene lists using DAVID bioinformatics resources. Nat Protoc 4:44-57. doi:10.1038/nprot.2008.211

4. Kanehisa M, Goto S (2000) KEGG: kyoto encyclopedia of genes and genomes. Nucleic Acids Res 28:27-30. doi:10.1093/nar/28.1.27

5. Lee JH, Gao C, Peng G, Greer C, Ren S, Wang Y, Xiao X (2011) Analysis of transcriptome complexity through RNA sequencing in normal and failing murine hearts. Circ Res 109:1332-1341. doi:10.1161/CIRCRESAHA.111.249433

6. Toischer K, Rokita AG, Unsold B, Zhu W, Kararigas G, Sossalla S, Reuter SP, Becker A, Teucher N, Seidler T, Grebe C, Preuss L, Gupta SN, Schmidt K, Lehnart SE, Kruger M, Linke WA, Backs J, Regitz-Zagrosek V, Schafer K, Field LJ, Maier LS, Hasenfuss G (2010) Differential cardiac remodeling in preload versus afterload. Circulation 122:993-1003. doi:10.1161/CIRCULATIONAHA.110.943431

Legends for video file

Online Movie I

Video-optical contractility analysis of a control EHT

Online Movie II

Video of a control EHT compared to an afterload enhanced EHT with inserted metal braces. Post deflection is markedly decreased due to increased afterload
